# Role of kidney injury in sepsis

**DOI:** 10.1186/s40560-016-0146-3

**Published:** 2016-03-23

**Authors:** Kent Doi

**Affiliations:** Department of Emergency and Critical Care Medicine, The University of Tokyo Hospital, 7-3-1 Hongo, Bunkyo, Tokyo, 113-8655 Japan

**Keywords:** Sepsis, Acute kidney injury, Chronic kidney disease, Lung injury, High mobility group box 1

## Abstract

Kidney injury, including acute kidney injury (AKI) and chronic kidney disease (CKD), has become very common in critically ill patients treated in ICUs. Many epidemiological studies have revealed significant associations of AKI and CKD with poor outcomes of high mortality and medical costs. Although many basic studies have clarified the possible mechanisms of sepsis and septic AKI, translation of the obtained findings to clinical settings has not been successful to date. No specific drug against human sepsis or AKI is currently available. Remarkable progress of dialysis techniques such as continuous renal replacement therapy (CRRT) has enabled control of “uremia” in hemodynamically unstable patients; however, dialysis-requiring septic AKI patients are still showing unacceptably high mortality of 60–80 %. Therefore, further investigations must be conducted to improve the outcome of sepsis and septic AKI. A possible target will be remote organ injury caused by AKI. Recent basic studies have identified interleukin-6 and high mobility group box 1 (HMGB1) as important mediators for acute lung injury induced by AKI. Another target is the disease pathway that is amplified by pre-existing CKD. Vascular endothelial growth factor and HMGB1 elevations in sepsis were demonstrated to be amplified by CKD in CKD-sepsis animal models. Understanding the role of kidney injury as an amplifier in sepsis and multiple organ failure might support the identification of new drug targets for sepsis and septic AKI.

## Introduction

Sepsis is defined by the Surviving Sepsis Campaign Guideline 2012 (SSCG 2012) as the presence (probable or documented) of infection together with systemic manifestations of infection [1]. Serum creatinine, a widely measured renal function marker, includes the definition by SSCG 2012 as an organ dysfunction variable with a serum creatinine increase of >0.5 mg/dL. A recent acute kidney injury (AKI) definition by the Kidney Disease: Improving Global Outcomes (KDIGO) includes increase in serum creatinine by 0.3 mg/dL within 48 h. Therefore, sepsis and AKI will be observed frequently in critically ill patients in ICUs. In addition, sepsis and AKI synergistically increase the mortality of ICU patients. No specific drug against sepsis and AKI is clinically available. Chronic kidney disease (CKD), defined as glomerular filtration rate (GFR) <60 mL/min/1.73 m^2^ for 3 months, is increasing all over the world because of not only the greater prevalence of obesity, diabetes, and hypertension but also improved longevity. The prevalence of CKD in ICUs is also increasing. In fact, CKD has recently been recognized as an important risk factor for AKI development and poor outcomes in sepsis. Complication of kidney injury worsens critical illness. Better management for kidney injury will improve the outcomes of sepsis.

## Review

### Epidemiology of kidney injury in ICU

AKI is a serious complication in critically ill patients because AKI strongly affects outcomes such as mortality and medical costs [2–4]. Recently, the KDIGO has defined diagnostic criteria and severity staging for AKI (Table 1) [5]. A recent meta-analysis involving 154 studies of more than 3,000,000 individuals revealed that one in five adults and one in three children worldwide developed AKI during a hospital episode of care [6]. The overall incidence of AKI in ICU patients ranges from 20 to 50 %. The severity of AKI was significantly associated with mortality [7]. It is noteworthy that dialysis-requiring AKI in ICU shows the highest mortality. Recent data from the Nationwide Inpatient Sample show a rapid increase of the incidence of dialysis-requiring AKI during the past decade in the USA [8].

**Table 1:** Definition and staging of AKI

Definition	AKI is defined as any of the following	
	1) Increase in SCr by >0.3 mg/dL within 48 h	
	2) Increase in SCr to >1.5 times baseline, which is known or presumed to have occurred within the prior 7 days	
	3) Urine volume <0.5 mL/kg/h for 6 h	
Severity	Serum creatinine	Urine output
Stage 1	1.5–1.9 times baseline, or	<0.5 mL/kg/h for 6–12 h
	>0.3 mg/dL increase	
Stage 2	2.0–2.9 times baseline	<0.5 mL/kg/h for >12 h
Stage 3	3.0 times baseline, or	<0.3 mL/kg/h for >24 h, or
	Increase in SCr to >4.0 mg/dL, or	Anuria for >12 h
	Initiation of renal replacement therapy	

*SCr* serum creatinine

CKD has also been recognized as a public health problem because its incidence and prevalence continue to increase, entailing poor outcomes and high costs [9, 10]. CKD is defined by a decreased estimated glomerular filtration rate (eGFR) calculated with age, gender, and by serum creatinine concentration (Table 2). In Japan, one in eight adults is estimated to be complicated with CKD, which is well known to contribute strongly to cardiovascular disease and high mortality [11]. Moreover, CKD, along with sepsis, is an important risk factor for AKI development [12]. Actually, CKD is found in approximately 30 % of AKI patients in the ICU [13, 14]. Hsu and colleagues reported that the odds ratios of AKI development were elevated progressively from 1.95 to 40.07 for stage 3 (45 < eGFR < 60) through stage 5 CKD (eGFR < 15) patients compared to patients with stage 1 and 2 CKD (eGFR > 60) [15]. Several observational studies found that 0.9–6.8 % of all patients admitted to the ICU have a prior diagnosis of end-stage renal disease (ESRD) [16]. Although the incidence of non-dialysis CKD in ICU has been poorly investigated, the prevalence of CKD in the ICU is assumed to be much higher than that of ESRD.

**Table 2:** Definition and staging of CKD

CKD is defined as either of the following present for >3 months					
1) Markers of kidney damage (one or more)					
	Albuminuria, urine sediment abnormalities, electrolyte, or other abnormalities attributable to tubular disorders, abnormalities detected by histology, structural abnormalities detected by imaging, history of kidney transplantation				
2) Decreased GFR; GFR <60 mL/min/1.73 m^2^ (GFR categories G3a–G5)					
GFR category	GFR (mL/min/1.73 m^2^)		Albuminuria category	AER (mg/day)	
G1	>90	Normal or high	A1	<30	Normal to mildly increased
G2	60–89	Mildly decreased	A2	30–300	Moderately increased
G3a	45–59	Mildly to moderately decreased	A3	>300	Severely increased
G3b	30–44	Moderately to severely decreased			
G4	15–29	Severely decreased			
G5	<15	Kidney failure			

*GFR* glomerular filtration rate, *AER* albumin excretion rate

### Pathophysiology of septic acute kidney injury

**Fig. 1:**
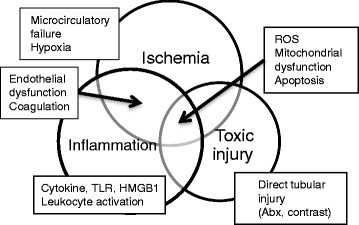
Pathophysiology of AKI. Three major areas of ischemia, inflammation, and direct toxic injury to the kidney contribute to the pathogenesis of AKI with significant overlap. Each mechanistic pathway identified by basic studies will be categorized into one of these major areas; however, some will lie simultaneously in two or three areas. Details are described in other review articles [71–73]. *ROS* reactive oxygen species, *TLR* toll-like receptor, *HMGB1* high mobility group box 1, *ABx* antibiotics

AKI is a syndrome with a broad spectrum of etiologies, and several mechanisms including ischemic/hypoxic, nephrotoxic, and inflammatory insults contribute to AKI development (Fig. 1). Depending on different clinical settings such as post-cardiac surgery, contrast media exposure, severe heart failure with low output, and sepsis, pathophysiology and clinical features of AKI will be different. Among these etiologies, sepsis is the leading cause of AKI in ICUs [14]. Reportedly, 45–70 % of all AKI is associated with sepsis [17–19]. Patients with both sepsis and AKI are widely recognized as having an unacceptably high mortality rate [17, 20]. Bagshaw and colleagues reported that in-hospital and ICU mortalities of septic AKI were increased, respectively, to 30 and 20 % and that higher mortality was observed across all the AKI severity categories [20].

Several pathophysiological mechanisms that are relatively specific for sepsis-induced AKI have been proposed (Table 3). Recent review articles discuss these mechanisms precisely [21–26], and a detailed description on this issue is beyond the aim of this review. Because of the complexity of sepsis and AKI, it should be noted that no single pathway can explain all the features of septic AKI. Each septic AKI patient moves along an individual disease trajectory. Therefore, the therapeutic targets vary with the underlying pre-existing conditions, time course, and disease trajectory of sepsis and AKI. Although many potential drug targets have been identified in animal models of sepsis and AKI, translation from animals to humans has been exceedingly difficult. The failure to translate results from animals to humans has been attributed to disease characteristics of sepsis and AKI (complexity and heterogeneity), inappropriate clinical trials, and animal models that do not fully mimic human sepsis [27].

**Table 3:** Potential pathophysiological mechanisms of septic AKI

Pro-inflammatory state	
Complement and coagulation activation	
Protease activation (heparan sulfate, elastase)	
Free radical formation	
Pro-inflammatory cytokine production (IL-1, IL-6, IL-18, TNF-α)	
Cell activation (neutrophil, macrophage, platelet, endothelial cell)	
Anti-inflammatory state	
Anti-inflammatory cytokine (IL-10)	
Reduced phagocytosis and chemotaxis	
Deranged immune function (lymphocyte apoptosis)	
Dysregulation of microcirculation	
Vasodilation-induced glomerular hypoperfusion	
Abnormal blood flow within the peritubular capillary network	

*TNF* tumor necrosis factor

### Remote organ injury induced by acute kidney injury

Dialysis-requiring AKI shows unacceptably high mortality of 40–50 % [28], with mortality increasing to 60–80 % when associated with distant organ dysfunction such as cardiac and respiratory failure [29, 30]. Remarkable progress has taken place in renal replacement therapy (RRT) in critical care. Therefore, uremic conditions of hemodynamically unstable patients in ICU can be treated successfully using continuous RRT (CRRT) [31]. Nevertheless, dialysis has not decreased mortality appreciably [8, 28, 32]. Although AKI in the ICU is associated with a high mortality, factors other than loss of kidney function appear to contribute to poor outcomes: non-dialysis-requiring AKI patients show considerably higher mortality than ESRD patients show [33].

Based on these observations, many basic researchers have started to elucidate the mechanisms of distant organ dysfunction caused by AKI [34]. The most investigated distant organ is the lung. Respiratory failure, which is frequently observed in septic patients, is caused by vascular leakage and subsequent pulmonary edema. Volume overload caused by AKI amplifies lung injury, but it can be prevented by removing excess extracellular fluid [35]. However, several clinical studies have implicated inflammation in the pathogenesis of lung injury complicated with AKI. For instance, elevated blood levels of inflammatory mediators such as plasminogen activator inhibitor-1, interleukin-6 (IL-6), and soluble tumor necrosis factor receptors are observed in ARDS patients complicated with AKI compared with non-AKI [36].

**Fig. 2:**
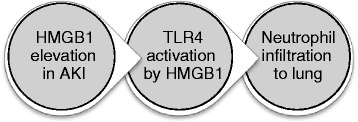
Possible pathway of lung injury induced by AKI. HMGB1 is a TLR4 agonist, and TLR4 induces inflammation including neutrophil activation. *TLR* toll-like receptor, *HMGB1* high mobility group box 1

Experimental studies using animal AKI models such as renal ischemia-reperfusion injury (IRI) and bilateral nephrectomy (BNx) have identified several different mechanisms by which AKI causes lung injury, including increased neutrophil infiltration, vascular permeability, dysregulation of salt and water transporters, and inflammatory cytokine and chemokine expressions [34, 37, 38]. Faubel and colleagues demonstrated that circulating IL-6 is a pathogenic mediator of lung injury in AKI [39, 40]. Toll-like receptor 4 (TLR4) plays fundamental roles in pathogen recognition and activation of innate immunity. TLR4 recognizes lipopolysaccharide (LPS), heparan sulfate, heat shock proteins, and high mobility group box 1 (HMGB1) [41]. Actually, HMGB1 has been shown to activate NF-κB by interacting with TLR4 on target cells [42]. BNx-induced lung injury characterized by neutrophil infiltration was partly reduced in TLR4-mutant C3H/HeJ mice, which is deficient in TLR4 signaling. Elevated blood HMGB1 levels were observed after BNx. Blockade of HMGB1 attenuated lung injury only in TLR4-wild type C3H/HeN mice. These observations suggest that TLR4–HMGB1 pathway contributes to lung injury induced by AKI (Fig. 2) [43].

### Amplification of multiple organ failure by pre-existing kidney injury

Epidemiological studies of human sepsis have demonstrated the importance of pre-existing comorbid conditions including CKD [44, 45]. Reportedly, patients with CKD have increased risk of morbidity and mortality from sepsis [46–48], although limited data are available for non-dialysis CKD patients [49]. These findings suggest that sepsis and septic AKI in clinical settings are remarkably influenced by underlying CKD. Star and colleagues established two-stage mouse models of pre-existing renal disease with subsequent sepsis (CKD-sepsis) to mimic the complexity of human sepsis [50, 51]. CKD was induced by 5/6 nephrectomy (5/6Nx) or folic acid injection. Evidence of CKD as reduced GFR and pathological renal injury such as glomerular sclerosis and interstitial fibrosis was observed 2 or 4 weeks after. Then, these CKD animals were subjected to cecum ligation and puncture (CLP) surgery, the most widely used animal model of sepsis [52, 53], which induces polymicrobial bacteremia and sepsis because of needle puncture of the ligated cecum, causing leakage of fecal contents into the peritoneum.

These CKD-sepsis models showed remarkably high mortality with increased blood levels of vascular endothelial growth factor (VEGF) and HMGB1. Although sepsis induced by CLP alone increased these mediators, CKD-sepsis animals showed significantly higher levels than non-CKD-sepsis did. It must be addressed that the CKD condition caused mild but significant VEGF and HMGB1 elevations before sepsis induction and acute complete loss of renal function by BNx also caused VEGF and HMGB1 elevations in blood. Importantly, VEGF neutralization with soluble fms-like tyrosine kinase 1 (sFLT-1) (a soluble VEGF receptor) and HMGB1-neutralizing antiserum attenuated other organ injury including the liver and lungs and improved the survival of CKD-sepsis animals. Taken together, pre-existing renal injury amplifies sepsis disease progression and sepsis-induced AKI by increasing VEGF and HMGB1.

The pro-inflammatory cytokine HMGB1 secreted from dying cells induces the release of other cytokines from macrophages and other cell types [54–56]. HMGB1 can induce the additional release of HMGB1 in RAW 264.7 cells [57]. Therefore, HMGB1 seems to amplify inflammation by positive feedback. Several basic studies have demonstrated that HMGB1 neutralizing therapy improves mortality of sepsis in non-CKD mice [57–59]. This treatment would work well in CKD-sepsis, which shows a more severe form of sepsis. Can we translate these findings on HMGB1 into clinical terms? In vitro analysis revealed that surface-treated polyacrylonitrile (AN69ST), which is now clinically available in Japan, shows a high capacity to adsorb HMGB1 [60]. Further investigation is necessary to elucidate the role of HMGB1 in human sepsis complicated with CKD.

### Perspectives for development of new treatment

Both sepsis and AKI have been recognized as a “graveyard for pharmaceutical companies” [61, 62] because no specific drug is currently available for these diseases in a clinical setting. Several new findings focusing on remote organ injury in AKI and amplification of septic reaction by CKD described above may suggest that humoral mediator removal would be effective in sepsis complicated with kidney injury. So far, efficient elimination of IL-6 and HMGB1 by blood purification technique has been reported. On the other hand, clinical trials that evaluated high-volume hemofiltration (HVHF) failed to show any protection even though HVHF could show significant removal of humoral mediators from the blood [63–65]. Another potential therapeutic strategy is mesenchymal stem cell (MSC) implantation. MSCs exhibit multiple beneficial properties by attenuating the inflammatory response, modulating immune cells, and promoting tissue healing [66–68]. MSCs are expected to home to sites of injury and use paracrine mechanisms to change the local environment to improve organ function and survival. Some study demonstrated the amelioration of sepsis-induced AKI by MSC administration [69, 70]. MSCs may be able to show their protective effects by regulating inflammatory cells and mediators with adaption to environmental changes induced by kidney injury complication.

## Conclusions

Complications of acute and chronic kidney injury are associated significantly with poor outcomes of sepsis. Although many epidemiological studies have already demonstrated these associations, the precise mechanisms by which kidney injury has a significant impact on other organs in sepsis remain unclear. Understanding the role of kidney injury as an amplifier in sepsis and multiple organ failure might enable the identification of new drug targets for sepsis and septic AKI.
